# Supplementary material for: Effect of Heterointerface on NO2 Sensing Properties of In-Situ Formed TiO2 QDs-Decorated NiO Nanosheets
Source: Nanomaterials (Basel). 2019 Nov 16;9(11):1628. doi: 10.3390/nano9111628 (PMC6915654; doi:10.3390/nano9111628)
Supplement: Supplementary file 1 [file nanomaterials-09-01628-s001.pdf]

nanomaterials

Supplementary

Effect of Heterointerface on NO_2 Sensing Properties of In-Situ Formed TiO_2 QDs-Decorated NiO Nanosheets

Congyi Wu [1,2], Jian Zhang [1], Xiaoxia Wang [1], Changsheng Xie [1], Songxin Shi [2,*] and Dawen Zeng [1,3,*]

[1] State Key Laboratory of Material Processing and Die & Mould Technology, School of Materials Science and Engineering, Huazhong University of Science and Technology (HUST), Wuhan 430074, China; wucongyi@163.com (C.W.); jian.zhang1@anu.edu.au (J.Z.); wxxjohn@163.com (X.W.); csxie@mail.hust.edu.cn (C.X.)

[2] State Key Lab of Digital Manufacturing Equipment and Technology, Huazhong University of Science and Technology (HUST), Wuhan 430074, China;

[3] Hubei Collaborative Innovation Center for Advanced Organic Chemical Materials, Hubei University, Wuhan 430062, China

* Correspondence: shisx@mail.hust.edu.cn (S.S.); dwzeng@mail.hust.edu.cn (D.Z.)

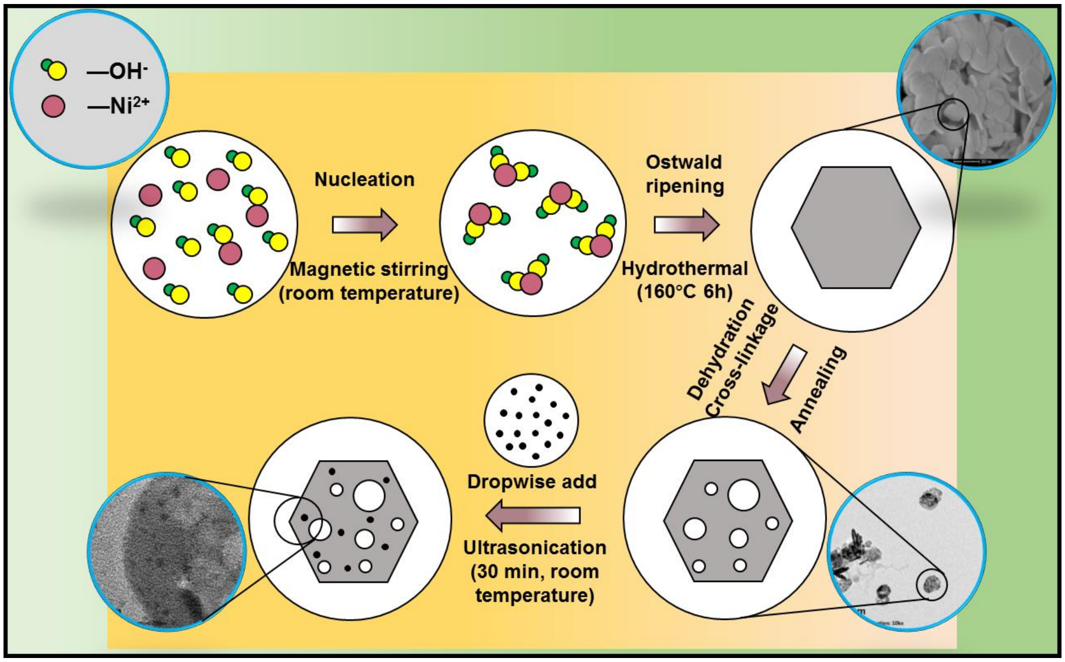

Figure S1. Schematic illustration of the synthesis procedure of the TiO_2-NiO nanocomposites.

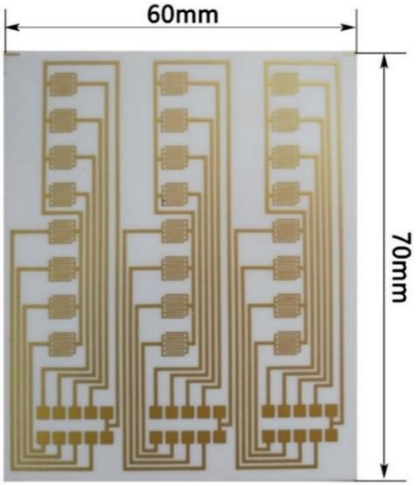

Figure S2. The schematic diagram of sensor substrate.

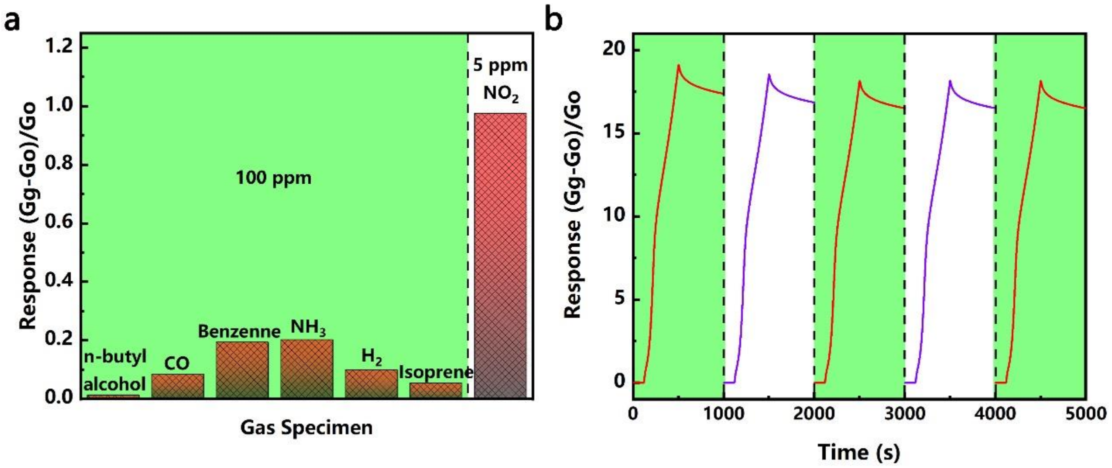

Figure S3. (a) The responses of the 5TiO$_2$QDs-NiO towards various gases (5 ppm for NO_2, 100 ppm for the rest). (b)The repeatability of the 5TiO$_2$QDs-NiO towards 60 ppm NO_2.

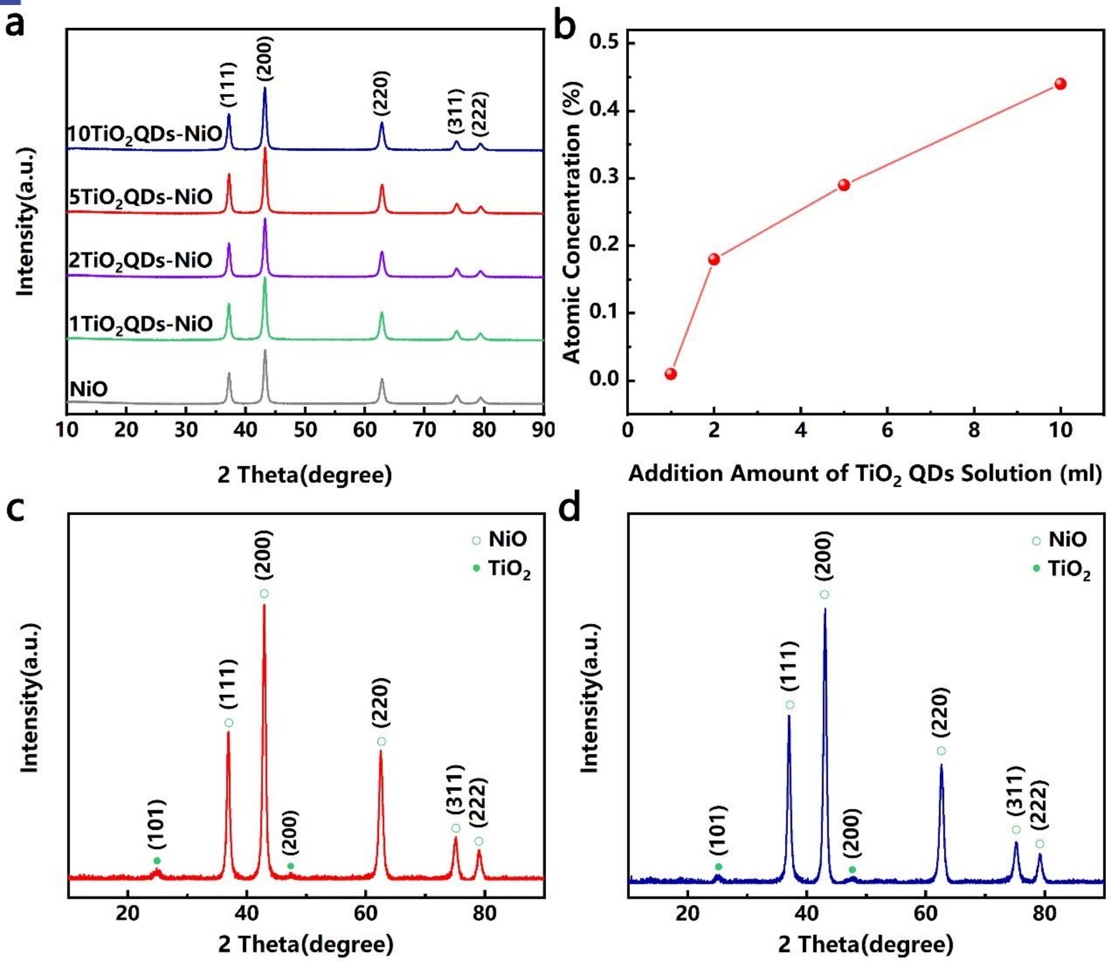

Figure S4. (a) XRD patterns of the NiO nanosheets and the TiO_2QDs-NiO nanohybrids; (b) Atomic concentration of Ti 2p of the TiO_2QDs-NiO nanohybrids; XRD patterns of (c) the 20$TiO_2$15-NiO and (d) the 50$TiO_2$30-NiO.

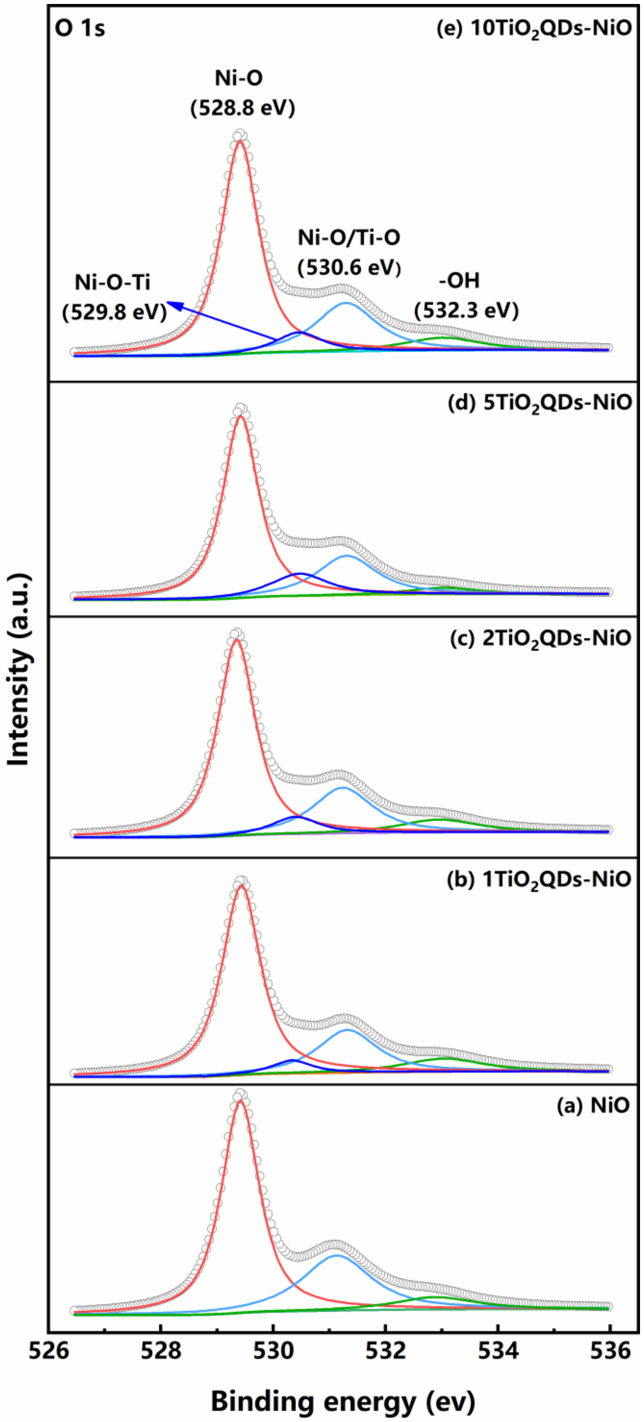

Figure S5. O 1s spectra of (a) the bare mesoporous NiO, (b) $1TiO_2QDs$-NiO, (c) 2 TiO_2QDs-NiO, (d) 5 TiO_2QDs-NiO, (e) 10 TiO_2QDs-NiO from XPS.

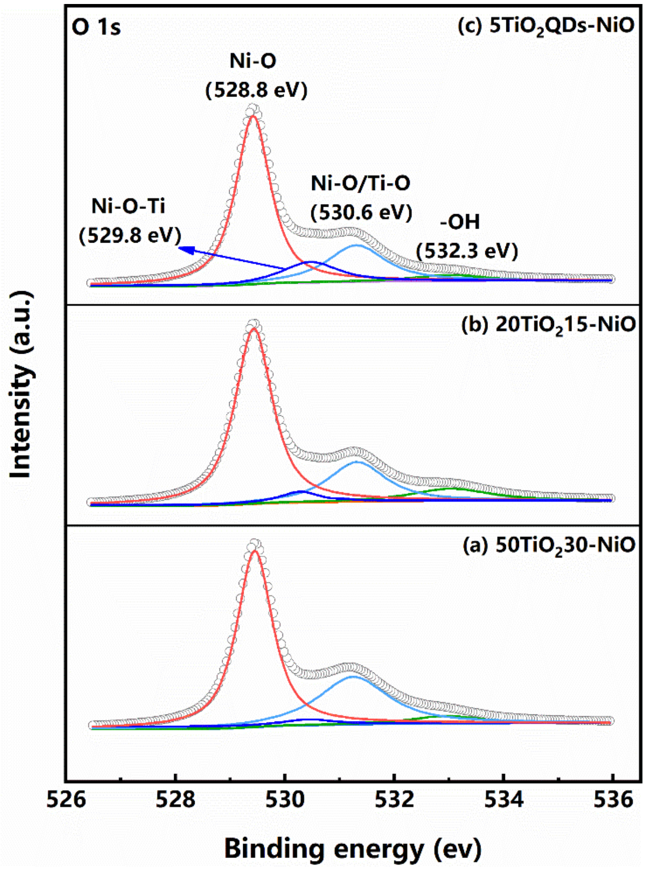

Figure S6. O 1s spectra of (a) 50TiO230-NiO, (b) 20TiO215-NiO, (c) 5 TiO2QDs-NiO from XPS.

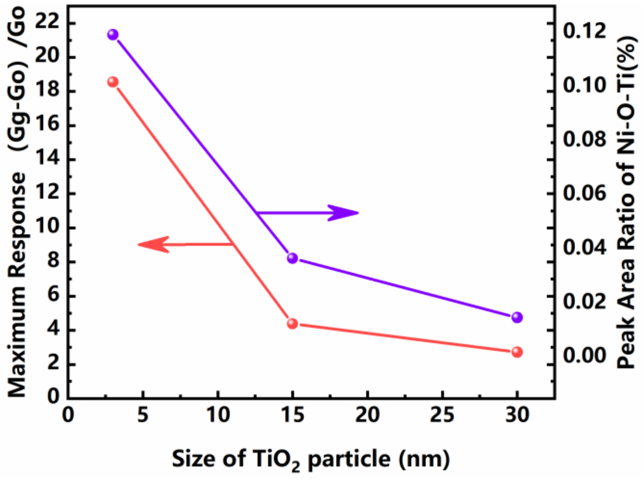

Figure S7. With the increase of TiO_2 nanoparticle size, comparison between the variation of the maximum responses to 60 ppm NO_2 and the variation of the peak area ratio of Ni-O-Ti.

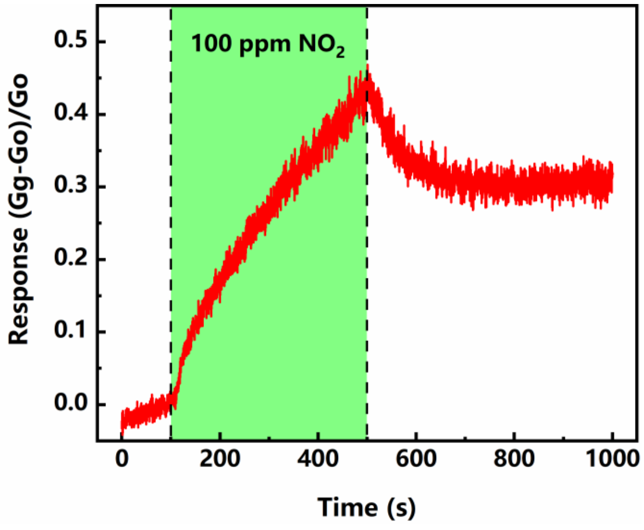

Figure S8. Dynamic sensitivity-recovery curves of TiO_2 QDs to 100ppm NO_2.

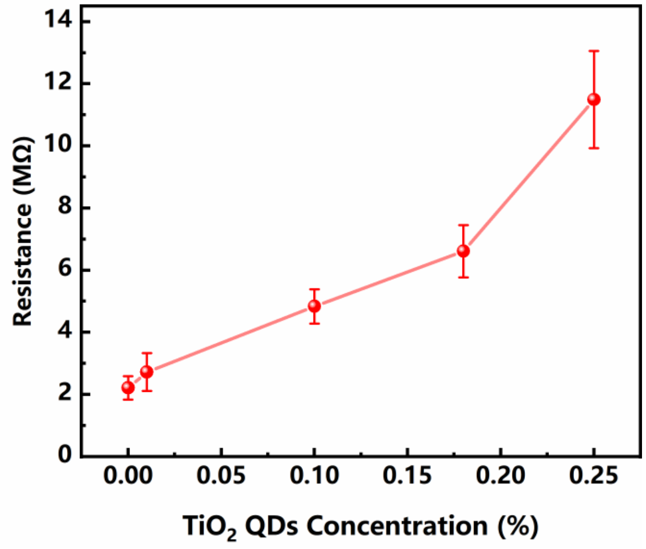

Figure S9. The Resistance of the nanohybrids with different addition quantity of TiO_2 QDs.
